# Protease-Activated Receptor 1 in Human Carotid Atheroma Is Significantly Related to Iron Metabolism, Plaque Vulnerability, and the Patient’s Age

**DOI:** 10.3390/ijms23126363

**Published:** 2022-06-07

**Authors:** Wei Li, Ehab Osman, Claes Forssell, Xi-Ming Yuan

**Affiliations:** 1 Obstetrics and Gynecology, Department of Biomedical and Clinical Sciences, Linköping University, 581 85 Linköping, Sweden; 2 Occupational and Environmental Medicine, Department of Health, Medicine and Caring Sciences, Linköping University, 581 85 Linköping, Sweden; ehab.osman82@gmail.com (E.O.); ximing.yuan@liu.se (X.-M.Y.); 3 Vascular Surgery, Linköping University Hospital, 581 85 Linköping, Sweden; clafors@gmail.com

**Keywords:** atherothrombosis, iron-related proteins, macrophages, PAR1

## Abstract

(1) Background: Protease-activated receptor 1 (PAR1) has regulatory functions in inflammation, atherogenesis, and atherothrombosis. Chronic iron administration accelerates arterial thrombosis. Intraplaque hemorrhage and hemoglobin catabolism by macrophages are associated with dysregulated iron metabolism and atherosclerotic lesion instability. However, it remains unknown whether expressions of PAR1 in human atherosclerotic lesions are related to plaque severity, accumulation of macrophages, and iron-related proteins. We investigated the expression of PAR1 and its relation to the expression of ferritin and transferrin receptors in human carotid atherosclerotic plaques and then explored potential connections between their expressions, plaque development, and classical risk factors. (2) Methods: Carotid samples from 39 patients (25 males and 14 females) were immunostained with PAR1, macrophages, ferritin, and transferrin receptor. Double immunocytochemistry of PAR1 and ferritin was performed on THP-1 macrophages exposed to iron. (3) Results: PAR1 expression significantly increases with the patient’s age and the progression of human atherosclerotic plaques. Expressions of PAR1 are significantly correlated with the accumulation of CD68-positive macrophages, ferritin, and transferrin receptor 1 (TfR1), and inversely correlated with levels of high-density lipoprotein. In vitro, PAR1 is significantly increased in macrophages exposed to iron, and the expression of PAR1 is colocalized with ferritin expression. (4) Conclusions: PAR1 is significantly related to the progression of human atherosclerotic lesions and the patient’s age. PAR1 is also associated with macrophage infiltration and accumulation of iron metabolic proteins in human atherosclerotic lesions. Cellular iron-mediated induction of PAR1 and its colocalization with ferritin in macrophages may further indicate an important role of cellular iron in atherothrombosis.

## 1. Introduction

Atherosclerosis-related cardiovascular disease (ACVD) is the leading cause of mortality and morbidity in many countries. Sudden disruption of an atherosclerotic plaque leads to platelet activation, atherothrombosis, and vessel occlusion. Atherothrombosis is the underlying condition that results in events leading to myocardial infarction, ischemic stroke, and vascular death [1].

Thrombin receptors, also known as protease-activated receptors (PRAs), are classically activated by thrombin and are critical in controlling the balance of hemostasis and atherothrombosis. PARs, including PAR1, are transmembrane G-protein-coupled receptors and are associated with the differentiation of human monocytes to macrophages to mediate regulatory functions in inflammation and atherogenesis [2,3]. Moreover, these protease-activated receptors are critical for the interplay between coagulation and inflammation in atherothrombosis. The expression of PAR1 increased in human atherosclerotic plaque [4,5], and inhibition of PAR1 attenuated the development of atherosclerotic lesions in the aorta and iliac arteries in apolipoprotein E–deficient (ApoE−/−) mice [6]. PAR1 inhibition may reduce thrombo-inflammatory event risks in patients with atherosclerosis independent of its effect on platelets [7]. However, clinicopathologic implications of PAR1 have not been investigated in human carotid atherosclerotic lesions.

Macrophage accumulation is the hallmark of atherosclerosis and plays a central role in atherosclerotic plaque initiation, progression, and atherothrombosis. Intraplaque hemorrhage and hemoglobin (Hb) catabolism by macrophages are associated with atherosclerotic lesion instability. We demonstrated that patients with advanced carotid atherosclerotic lesions had significantly higher levels of Hb in atherosclerotic lesions and accumulation of CD68-positive macrophages was significantly associated with iron-related proteins, ferritin, TfR1, and the development and severity of human carotid plaques [8,9]. In an animal model, it was shown that a thrombin injection-induced hemorrhage in the ipsilateral hemisphere was determined by Perls’ staining for iron and by measuring brain Hb [10]. However, it remains unknown whether the expressions of PAR1 in human atherosclerotic lesions are related to plaque severity, accumulation of macrophages, and iron metabolism-related proteins.

In the present study, we investigated the expression of PAR1 and its relation to the expression of ferritin and TfR in human carotid atherosclerotic plaque and then explored potential connections between their expressions, plaque development, and some established risk factors. We also investigated the role of cellular iron in PAR1 expression and its relation to the expression of ferritin in macrophages in vitro.

## 2. Results

### 2.1. PAR1 Expression Is Significantly Increased with the Progression of Human Atherosclerotic Plaques and Patient’s Age

**Figure 1:**
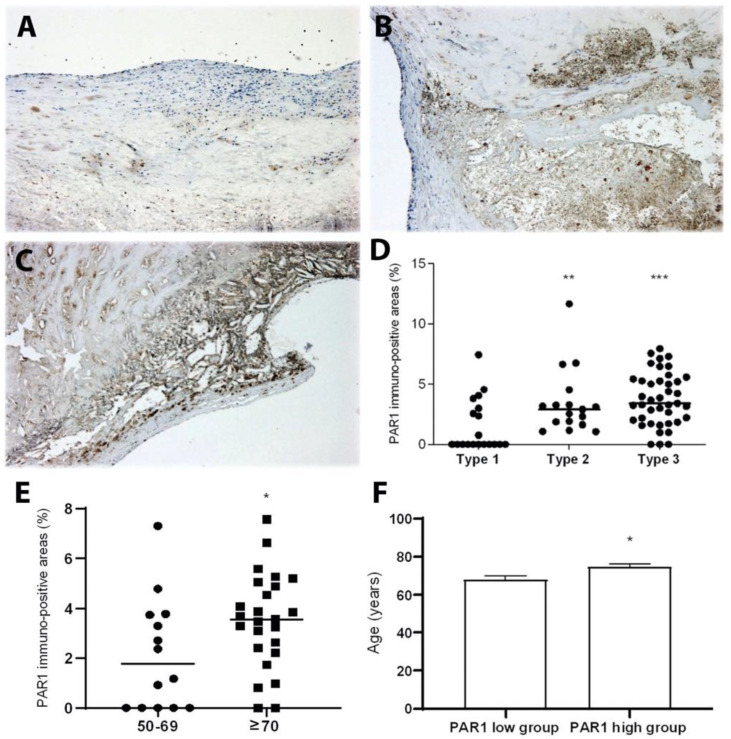
PAR1 significantly increased in advanced carotid atherosclerotic lesions, particularly lesions of elderly patients. (**A**–**D**) PAR1 expression in early and advanced atherosclerotic plaques. Representative images of type 1 (**A**), type 2 (**B**), and type 3 (**C**) lesions. (**D**) Image analyses of PAR1 in type 1 (n = 19), type 2 (n = 17), and type 3 (n = 41) lesions, ** *p* < 0.01 and *** *p* < 0.001 vs. type 1. (**E**,**F**) Expression of PAR1 in carotid atherosclerotic lesions increased in elderly patients. (**E**) Plaques from patients at age > or = 70 years (n = 25) had significantly higher levels of PAR1 compared to the ones at age < 70 (n = 14), * *p* < 0.05. (**F**) Patients in the group with higher levels of PAR1 were significantly older than the ones from the group with lower levels of PAR1. The plaques with PAR1 levels ≤ median are defined as the PAR1 low group (n = 20), while the ones with PAR1 levels > median are defined as the PAR1 high group (n = 19). The patients with higher levels of PAR1 were significantly older than the ones with lower levels of PAR1, * *p* < 0.05 vs. patients in the PAR1 low group.

As compared to early lesions—type 1 (Figure 1A), the expression of PAR1 was more frequent in the advanced plaques (Figure 1B,C). The quantitative analysis showed that the levels of PAR1 significantly increased in type 2 (plaques with necrotic cores) and type 3 plaques (ruptured plaques) compared to type 1 plaques (Figure 1D). In the non-lesion areas, there was no detectable level of PAR1.

To assess possible interactions between thrombosis and increased age, a well-established risk factor in ACVD, the immunopositive areas of PAR1, were compared among patients between the ages of 50 and 69 or ≥70 years. We chose 70 as a mid-point because the mean ages were above 70 years for all patients (Supplementary Table S1). We found that lesions from patients at age ≥70 had significantly increased expressions of PAR1 (Figure 1E). The association of between PAR1 levels in and patients ages was further analyzed by dividing levels of PAR1 into low or high expression groups using the median value of PAR1, namely the PAR1 low group (≤median) and PAR1 high group (>median). As shown in Figure 1F, patients with higher levels of PAR1 in the lesions were significantly older than the ones with lower levels of PAR1. In contrast, diabetes mellitus, hypertension, statin treatment, and gender had no significant influence on the expression of PAR1. Moreover, PAR1 expression levels in the lesions from patients over age 70 were not significantly associated with diabetes mellitus, hypertension, and statin treatment. Moreover, PAR1 expression in human carotid plaque was significantly correlated with the patient’s age (sr = 0.41, *p* < 0.01).

### 2.2. PAR1 Expression Is Significantly Correlated with Accumulation of CD68-Positive Macrophages, Ferritin, and TfR1, and Inversely Correlated with Levels of HDL

Earlier, we showed that TfR1 (a major iron importer) and ferritin (iron storage and stress protein) are highly expressed in CD68-positive macrophages and are associated with instability and ruptured human carotid plaque [8,9].

**Figure 2:**
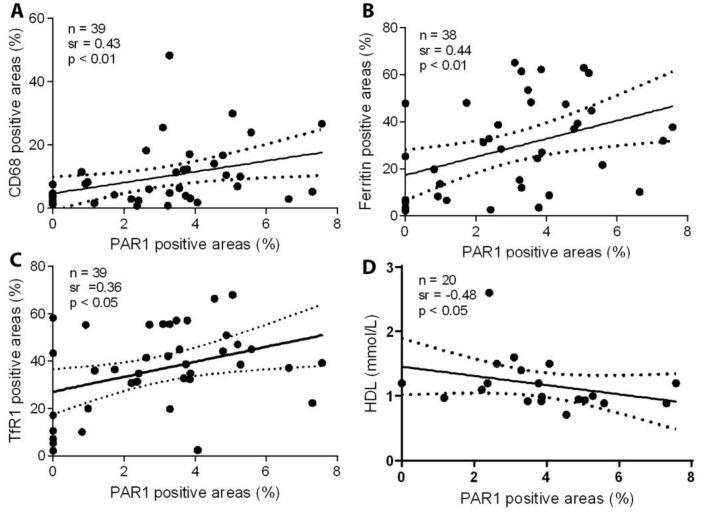
The expression of PAR1 in carotid atherosclerotic lesions was significantly correlated with levels of CD68-positive macrophages, ferritin, and TfR1, and inversely correlated with serum HDL. Serial sections of human carotid plaques were immunostained with antibodies against PAR1, CD68, ferritin, or TfR1, and images of the immunohistochemistry were analyzed as described in the Methods section. The expression of PAR1 was significantly correlated with CD68 (**A**), ferritin (**B**), and TfR1 (**C**), and was inversely correlated with HDL (**D**), as assessed by Spearman’s correlation coefficient test.

Here, we further examined the expression of PAR1 in relation to CD68 macrophages, ferritin, and TfR1. In the serial sections of all studied carotid lesions, the expression of PAR1 was significantly correlated with macrophage infiltration (Figure 2A), ferritin accumulation (Figure 2B), and TfR1 expression (Figure 2C). An analysis of serial cross-sections from carotid atherosclerotic lesions revealed no correlation between levels of PAR1 and actin-positive areas for smooth muscle cells. The expression of PAR1 was significantly and inversely correlated with levels of HDL (Figure 2D).

### 2.3. PAR1 Is Significantly Increased in Macrophages Exposed to Iron and Colocalized with Ferritin Expression

**Figure 3:**
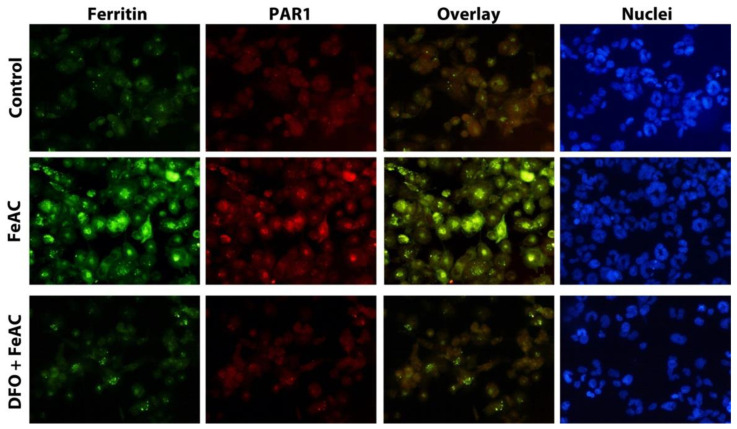
Iron exposure causes significant induction of cellular ferritin and PAR1 in THP-1 macrophages. THP-1 macrophages were either left untreated or were treated with FeAC (100 µg/mL) for 24 h or pretreated with iron chelator DFO (1 mM) for 1 h and then exposed to FeAC for 24 h without DFO. Following double immunocytochemistry for PAR1 and ferritin, the cells were examined by fluorescence microscopy. Representative photographs of PAR1 (red) and ferritin (green). Nuclei stained blue with DAPI.

Intraplaque hemorrhage and Hb catabolism by macrophages are associated with iron accumulation in the form of ferritin in the progression of atherosclerotic lesions. We reported earlier that under inflammatory conditions, iron may be exocytosed by macrophages that previously had their lysosomal apparatuses enriched with iron due to erythrophagocytosis following intraplaque hemorrhages [11]. However, it is unknown whether PAR1 expression in macrophages is iron exposure-related. To study the effect of cellular iron on PAR1 expression, THP-1 macrophages were exposed to 100 µg/mL FeAC and the expression of PAR1 and ferritin was examined. As seen in Figure 3, iron exposure resulted in the induction of both ferritin and PAR1, which were colocalized. The iron exposure mediated induction of ferritin and PAR1 was clearly averted by treatment of iron chelator deferoxamine (DFO, 1 mM) (Figure 3).

## 3. Discussion

PAR1, involved in atherothrombosis and inflammation, increased in human atherosclerotic lesions; and inhibition of PAR1 attenuates the development of atherosclerotic lesions in mice [4,5,6]. However, it is unknown whether PAR1 is related to human plaque severity and plaque rupture. In the present study, we demonstrated that PAR1 expression increases with the patient’s age and progression of human atherosclerotic plaque. Furthermore, PAR1 expression in atherosclerotic plaque is significantly associated with ferritin and TfR1 in macrophages; the association between cellular iron and PAR1 expression was verified in a macrophage model.

Thrombin activates cellular PARs and modulates multiple processes in the vascular system, including increased vascular permeability, inflammation, and neovessel formation. These cellular effects of thrombin are mediated by its receptor, PAR-1, widely distributed in platelets, endothelial cells, and macrophages. PARs are key receptors in the pathogenesis of atherothrombosis [12]. Our results on the associations between PAR1 and the severity or rupture of human carotid plaque further reveal that PAR1 may contribute to the progression of human atherosclerotic lesions. This is an age-related process in atherogenesis since PAR1 expression is significantly increased with increases in the patient’s age.

Our results show that there are increased protein levels of PAR1 in human carotid plaques, and we have no information on whether the expression of PAR1 in human lesions is thrombin-dependent. However, it is already known that PAR-1 is irreversibly activated by several factors, such as thrombin, tissue factor, endothelial protein C receptor, metalloproteases (MMPs), and bacteria [13]. In human atherosclerotic lesions, elevated expressions of these factors—thrombin [14], MMPs [15], and bacteria [16]—have been demonstrated. We speculate that PAR1 in human atheroma is irreversibly activated by the above factors and may be partially thrombin-dependent. Interestingly, recent immunofluorescence experiments revealed colocalization of PAR1 with toll-like receptors (TLRs)—TLR2 and TLR4—in human carotid atherosclerotic lesions, pointing to activation of TLRs and interaction with PAR1 in an innate immune response in carotid atherosclerosis [7].

We further investigated why PAR1 expression is associated with cellular iron metabolism in the form of increases in the expression of ferritin and TfR1. Iron accumulation in tissues has been implicated in several chronic diseases, including ACVD. Chronic iron loading markedly accelerates thrombus formation after arterial injury, increases vascular oxidative stress, and impairs vasoreactivity [17]. The source of cellular iron in the interaction between PAR1 and atherothrombosis is most likely from Hb catabolism by macrophages due to erythrophagocytosis following intraplaque hemorrhage in atherosclerosis, as we have proposed in 1996 [18].

Iron accumulation may contribute to oxidative stress. Several previous studies, including some of ours, have demonstrated that the expression of various oxidative stress markers in human atheroma lesions is associated with plaque iron accumulation. We earlier reported that compared with normal arterial tissues, there are substantial and simultaneous increases in levels of both cellular iron and ceroid in human carotid atherosclerotic lesions [19]. The ceroid or lysosome-derived lipopigment is derived from oxidative damage of proteins and lipids [20]. Moreover, oxidation products, such as aldehydes in a soluble aldehyde-rich fraction, carbonyls, and lipid hydroperoxides in an insoluble ceroid-like substance, have been considered major toxic components in the atheroma material [19]. Recently, using a proteomic analysis of human carotid atherosclerotic lesions, we demonstrated that there is a lesion-dependent expression of iron-associated proteins (ferritin, hemopexin, and serotransferrin) and oxidative stress-related proteins (glutathione transferase and peroxiredoxin-1) [21]. Using synchrotron radiation-induced X-ray fluorescence, iron was identified in both symptomatic and asymptomatic plaques. In acutely symptomatic plaque, iron is found within the thrombus in the presence of macrophages. The abundance of iron in symptomatic plaque is associated with the source patient’s LDL cholesterol [22].

Here, for the first time, we demonstrate that levels of ferritin and TfR1 are correlated with the expression of PAR1 in human atherosclerotic lesions, which further supports the role of cellular iron in atherothrombosis. Our in vitro experiments on the macrophage model confirmed the association between cellular iron metabolism and PAR1 expression, which is consistent with a previous study that described that the thrombin receptor was primarily located in endosomal compartments and colocalized with the transferrin receptor in human endothelial cells [23].

There is no report on a relationship between PAR1 in human atherosclerotic lesions and levels of HDL. Our data on the inverse correlation between them indicate proatherogenic effects of PAR1 may be related to low levels of HDL-C in atherosclerosis. The result is preliminary, and while intriguing, it requires confirmation in a large-scale study. It is known that certain subspecies of HDL may act as natural antioxidants preventing oxidation of low-density lipoprotein and biological membranes. The antioxidant function may be attributed to the inhibition of synthesis or neutralization of free radicals and reactive oxygen species by HDL and associated enzymes to transfer oxidation-prone lipids from LDL and biological membranes to HDL for catabolism [24]. The low HDL level and its association with PAR1 expression demonstrated in our study may well indicate a potential role of HDL in counteracting oxidative status-related induction of PAR1 in atherosclerosis.

### Limitation

The present study mainly focused on the pathology of human carotid atherosclerotic lesions. These carotid lesions were obtained due to either clinical symptoms or as preventative measures for strokes. Thus, the study is only indicative of possible risks of plaque instability and rupture. The sample sizes were small, resulting in clear variations of tissue expression levels; a larger prospective study is needed to confirm the findings of our study. In vitro experiments only confirmed a possible link between cellular iron metabolism and PAR1 observed in human atherosclerotic lesions; further studies on signaling mechanisms underlying the interaction between the PAR1 system and iron metabolism following erythrophagocytosis are needed. Moreover, it remains unknown whether the iron-dependent increase in PAR1 expression is specific to macrophages, which calls for further studies to investigate PAR1 expression in different types of cells following differential iron overloading conditions.

## 4. Materials and Methods

### 4.1. Collection of Carotid Artery Samples

The atherosclerotic carotid arteries were collected from patients who underwent carotid endarterectomies at Linköping University Hospital. This study was approved by the Regional Ethical Review Board in Linköping (03-499, 2003) and all methods were performed in accordance with the relevant guidelines and regulations. Written informed consent was obtained from all participants.

Carotid samples from 39 patients (25 males and 14 females) were included in the present study. Patients with no neurological symptoms six months prior to the operation were designated as asymptomatic (Asymp, n = 4), whereas patients with transitory ischemic attacks, minor stroke, or amaurosis fugax were considered symptomatic (Symp, n = 35). Several stroke risk factors, including age, hypertension (defined by hypertension history and diastolic blood pressure ≥90 mmHg, all received blood pressure-lowering treatment), smoking (defined as regular smoking >5 years), and diabetes mellitus (defined as regular administration of diabetes medication) were analyzed, which did not show statistical differences between asymptomatic and symptomatic patients (Supplemented Table S1).

Carotid artery samples were collected immediately post-endarterectomy and fixed in 4% formaldehyde. Three to five cross-sectional segments of each specimen were embedded in paraffin.

### 4.2. Immunohistochemistry

Paraffin-embedded carotid arteries were deparaffinized in xylene, rehydrated in graded ethanol, and subjected to immunostaining. Immunohistochemistry was performed on serial sections, as described previously [9]. The primary antibodies used were macrophage marker CD68 clone PG-M1 (Dako, Glostrup, Denmark), smooth muscle actin (SMC) clone 1A4 (Dako), ferritin, TfR (Dako), and thrombin receptor (protease-activated receptor 1, Sigma, Saint Louis, USA). The immunoreactions were visualized using the EnVision+/horseradish peroxidase (Dako) method and ChemMate EnVision Detection Kit (Dako). Control sections without primary antibodies or with non-immune IgG were run for each protocol, resulting in consistently negative results. The slides were counterstained with hematoxylin.

All histological sections were examined under a light microscope, and the images were digitalized with the Image Grabber program (Toronto, ON, Canada). The microscope was set on the same parameters used to scan all samples. The randomly digitalized images were analyzed with Adobe Photoshop (v5.5) as described previously [9]. The individual responsible for the analysis was blinded to patient information.

### 4.3. Classification of the Plaques

To investigate whether the expression of PAR1 was related to plaque progression, all carotid artery samples were classified into three groups based on their morphology and plaque components, as described previously [9]. In brief, the plaques were classified into early and advanced plaques. Early lesions (type 1) were intact lesions without necrotic cores. Advanced lesions were defined as intact plaques (type 2, with an intact fibrous cap, necrotic core formation, and inflammatory cell accumulation) or ruptured plaques (type 3, with a ruptured fibrous cap, often containing a large necrotic core, cholesterol crystals, internal plaque hemorrhage, or thrombosis).

### 4.4. Cell Cultures and Experimental Conditions

The THP-1 monocytic cell line was obtained from American Type Culture Collection (ATCC, Rockville, MD, USA) and cultured in RPMI 1640 medium (Invitrogen, Waltham, CA, USA), supplemented with 10% fetal bovine serum (Invitrogen) and 1% penicillin-streptomycin (Invitrogen). The cells were sub-cultured twice a week. THP-1 cells were differentiated into macrophages by incubating with phorbol 12-myristate 13-acetate (50 µM for 24 h). Differentiated macrophages were used for experiments after incubation in normal media for two days. In the experiments, the cells were exposed to 100 µg/mL ferric ammonium citrate (FeAC) for 24 h or pretreated with 1 mM iron chelator deferoxamine (DFO) for 1 h and then exposed to FeAC for 24 h without DFO in the culture media. Untreated cells were used as controls.

### 4.5. Double Immunocytochemistry

To investigate whether iron exposure can induce a simultaneous expression of PAR1 and ferritin, double immunocytochemistry of ferritin and PAR1 was performed. Cells were fixed in 4% paraformaldehyde at 4 °C and permeabilized with 0.1% saponin. The cells were incubated with anti-PAR1 overnight at 4 °C followed by incubation with Alexa fluor goat anti-rabbit antibodies (Invitrogen) for 1 h at room temperature. The cells were then incubated with FITC conjugated ferritin for 1 h at room temperature. All immune-stained cells were mounted with DAPI-containing mounting media (Vector Laboratories, Inc., Newark, CA, USA) and analyzed with immunofluorescence microscopy using the 40× oil-immersion objective.

### 4.6. Statistical Analysis

Continuous data are expressed as mean ± SEM. Differences were compared by a Kruskal–Wallis test for multiple groups, the Mann–Whitney U test for two groups, and chi-square for a comparison of categorical data. Spearman’s correlation test was used to examine the correlations among PAR1, CD68, ferritin, TfR1, and high-density lipoprotein (HDL). The results are presented as Spearman’s correlation coefficient (r). *p* ≤ 0.05 was considered statistically significant.

## 5. Conclusions

PAR1 is significantly related to plaque progression in human atherosclerotic lesions and the patient’s age. PAR1 is also associated with macrophage infiltration and accumulation of iron metabolic proteins in human atherosclerotic lesions. Cellular iron-mediated induction of PAR1 and its colocalization with ferritin in macrophages may further indicate an important role of cellular iron in atherothrombosis.

## Data Availability

The data presented in this study are available from the corresponding author upon reasonable request.

## Supplementary Materials

The following supporting information can be downloaded at: https://www.mdpi.com/article/10.3390/ijms23126363/s1.
