# Supplementary material for: Protease-Activated Receptor 1 in Human Carotid Atheroma Is Significantly Related to Iron Metabolism, Plaque Vulnerability, and the Patient’s Age
Source: Int J Mol Sci. 2022 Jun 7;23(12):6363. doi: 10.3390/ijms23126363 (PMC9223560; doi:10.3390/ijms23126363)
Supplement: Supplementary file 1 [file ijms-23-06363-s001.zip › ijms-1734764-supplementary.pdf]

Supplemented Table S1. Basic clinical information

	Total	Asymptomatic	Symptomatic	
n	39	4	35	p
Ages, y ± SE	71.1 ± 1.5	78.0 ± 1.9	70.3 ± 1.6	ns
Sex, male (%, n)	64.1 (25)	75.0 (3)	62.8 (22)	ns
Diabetes mellitus, % (n)	12.8 (5)	25 (1)	11.4 (4)	ns
Hypertesion, % (n)	74.3 (29)	50.0 (2)	77.1 (27)	ns
Smoking, % (n)	41.0 (16)	0 (0)	45.7 (16)	ns
Statin treatment, % (n)	46.1 (18)	25.0 (1)	48.6 (17)	ns
Total cholesterol	5.01 ± 0.3	5.2 ± 0.6	4.98 ± 0.3	ns
LDL	2.79 ± 0.2	2.67 ± 0.74	2.81 ± 0.2	ns
HDL	1.20 ± 0.09	1.70 ± 0.47	1.12 ± 0.06	ns
TG	2.04 ± 0.2	1.87 ± 0.35	2.07 ± 0.23	ns
Hb	142 ± 1.89	141 ± 13.02	142 ± 1.83	ns
MCV	92.4 ± 0.7	95.0 ± 2.5	92.2 ± 0.7	ns
MCHC	330 ± 1,8	332 ± 5.1	330 ± 1.9	ns
EVF	43.2 ± 0.6	42 ± 3.5	43.3 ± 0.6	ns
